# Treatment of Primary Solid Renal Tumours Using Histotripsy: Study Protocol for the CAIN Feasibility Trial

**DOI:** 10.1007/s00270-025-04035-5

**Published:** 2025-04-28

**Authors:** Tze Min Wah, Joseph F. Amaral, Paul F. Laeseke

**Affiliations:** 1 Diagnostic and Interventional Radiology, Leeds Teaching Hospitals Trust and Leeds Institute of Medical Research, University of Leeds, Leeds, West Yorkshire UK; 2 Warren Alpert Medical School of Brown University, Providence, Rhode Island, USA; 3 Department of Radiology, School of Medicine and Public Health, University of Wisconsin, Madison, WI USA; 4 Department of Biomedical Engineering, University of Wisconsin - Madison, Madison, WI USA

**Keywords:** Image guided ablation, Ultrasound, Histotripsy, Non-thermal ablation, Kidney cancer, Technical success, Complications

## Abstract

**Purpose:**

The purpose of this prospective, multi-centre, single-arm feasibility trial is to evaluate the technical success and safety profile of the HistoSonics System for the treatment of primary solid renal tumours using histotripsy.

**Methods/design:**

The CAIN trial will enrol up to 20 patients with a non-metastatic solid renal tumour ≤ 3 cm treated with histotripsy. Histotripsy is a non-thermal and non-ionizing mechanical process of tissue destruction resulting from the targeted delivery of focused ultrasound pulses. The primary endpoints are technical success and freedom from index procedure related major complications. Technical success is defined as complete coverage of the tumour as determined ≤ 36 h post-index procedure by contrast-enhanced magnetic resonance imaging (MRI) or computerized tomography (CT). An index procedure related major complication is defined as Clavien-Dindo Classification Grade 3 or higher up to 30 days after the last histotripsy procedure. Treated patients will have a follow-up visit performed at 14 days, 30 days, 90 days, and 180 days post-procedure.

*Trial Registration*: Clinicaltrials.gov identifier NCT05432232 & NIHR CRN CPMS 53429.

**Graphical Abstract:**

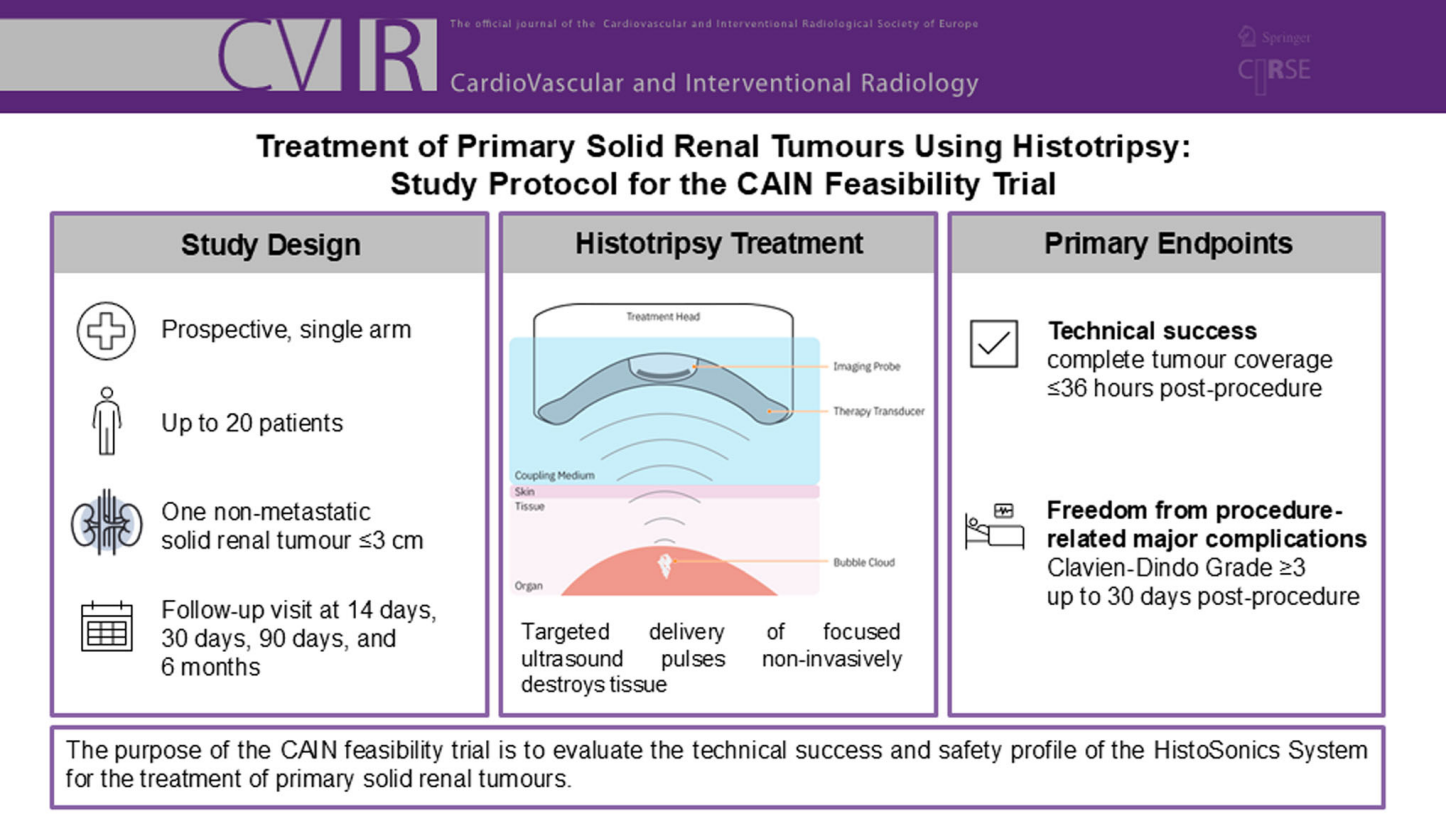

## Introduction

Renal cell carcinoma (RCC) accounts for 2% of global cancer diagnoses and deaths, with an estimated 403,000 people diagnosed with neoplasms of the kidney in 2018, according to GLOBOCAN data [1]. The incidence of renal cancer has more than doubled in the developed world over the past half-century in large part due to better and more frequent abdominal imaging. Over 50% of renal cell carcinomas are discovered incidentally during abdominal imaging for other reasons [1].

The latest guidelines from the American Urological Association and National Comprehensive Cancer Network recommend partial nephrectomy as the standard of care for small renal masses (4 cm; clinical stage T1a), but also endorse thermal ablation, active surveillance, and radical nephrectomy as acceptable alternatives for selected patients with comorbidities and preferences [2, 3]. However, thermal ablation procedures have limitations, particularly for treating central tumours, including the need to avoid major renal vessels and other critical structures (e.g., renal pelvis and ureter), limitations in targeting, inability to accurately monitor therapy during treatment, and risk of tumour seeding in the percutaneous tract [4].

Given the indolent nature of T1 renal carcinoma, the high prevalence in older age groups at higher risk of surgical procedures, and limitations of thermal ablation, a less invasive, non-thermal approach to destroy renal tumours is needed. The HistoSonics Investigational System (HistoSonics System) is an image-guided device designed to deliver non-invasive, non-thermal histotripsy for local treatment that has the potential to overcome many limitations of other focal kidney tumour treatment options. Histotripsy mechanically destroys targeted tissue through the precise targeting of acoustic cavitation [5–7]. Preclinical studies have shown that histotripsy can destroy normal renal tissue safely and effectively in a porcine model without damage to adjacent structures. In addition, these studies also demonstrated a relative preservation of the collecting system and fewer bleeding events when compared with cryoablation [8–10]. The technology received marketing authorization from the United States Food and Drug Administration in October 2023 for the destruction of liver tumours, based on the results of the #HOPE4LIVER trials which demonstrated successful non-invasive destruction of liver tumours using histotripsy [11]. The purpose of the CAIN feasibility trial is to evaluate the technical success and safety profile of the HistoSonics System for the treatment of primary solid renal tumours.

## Methods

### Trial Design

CAIN is a prospective, multi-centre, single-arm pilot trial; total enrolment will be up to twenty (20) patients, at up to four (4) clinical sites in Europe and/or United Kingdom. The trial is funded and sponsored by HistoSonics, Inc. (Plymouth, MN, USA) and is managed by IQVIA MedTech NV (Berchem, Belgium). The CAIN trial was designed to generate data to support a pivotal trial for the HistoSonics System for use in the kidney.

A Clinical Events Committee (CEC) was established to adjudicate adverse events reported during the trial; the CEC is not affiliated with the sponsor or actively participating in the trial. The patient’s clinical data is entered via electronic case report forms into iMedNet (MedNet Solutions, Inc., Minnetonka, MN, USA). The imaging assessments are uploaded and managed by an independent imaging core lab, Intrinsic Imaging LLC (Bolton, MA, USA), who was selected to perform quality control and independent review of images to minimize confirmation bias.

### Histotripsy Device

In the CAIN trial, the HistoSonics System, manufactured by HistoSonics, Inc. (Plymouth, MN, USA), is being studied for the non-invasive destruction of kidney tissue using histotripsy, a non-thermal mechanical process of focused ultrasound.

Histotripsy is based on the delivery of acoustic energy in the form of short (< 50 microseconds) very high intensity pulses which induces controlled cavitation to mechanically homogenize targeted tissue [5]. Cavitation occurs when a sufficiently negative pressure is applied to a fluid or tissue to cause microbubble formation from fluid vaporization and release of dissolved gas [12]. Microsecond, high-pressure pulses applied by an ultrasound transducer outside the body and focused on the target tissue are used to generate a cluster of microbubbles. Once formed, the microbubbles exhibit highly dynamic patterns of oscillation and inertial collapse which impart severe stress and strain on surrounding cells and tissues to produce cellular and tissue destruction at the target [7]. With sufficiently high pressure and adequate number of pulses, the target tissue can be completely destroyed, as demonstrated pre-clinically, creating a fluid homogenate with no recognizable cellular structures.

**Fig. 1:**
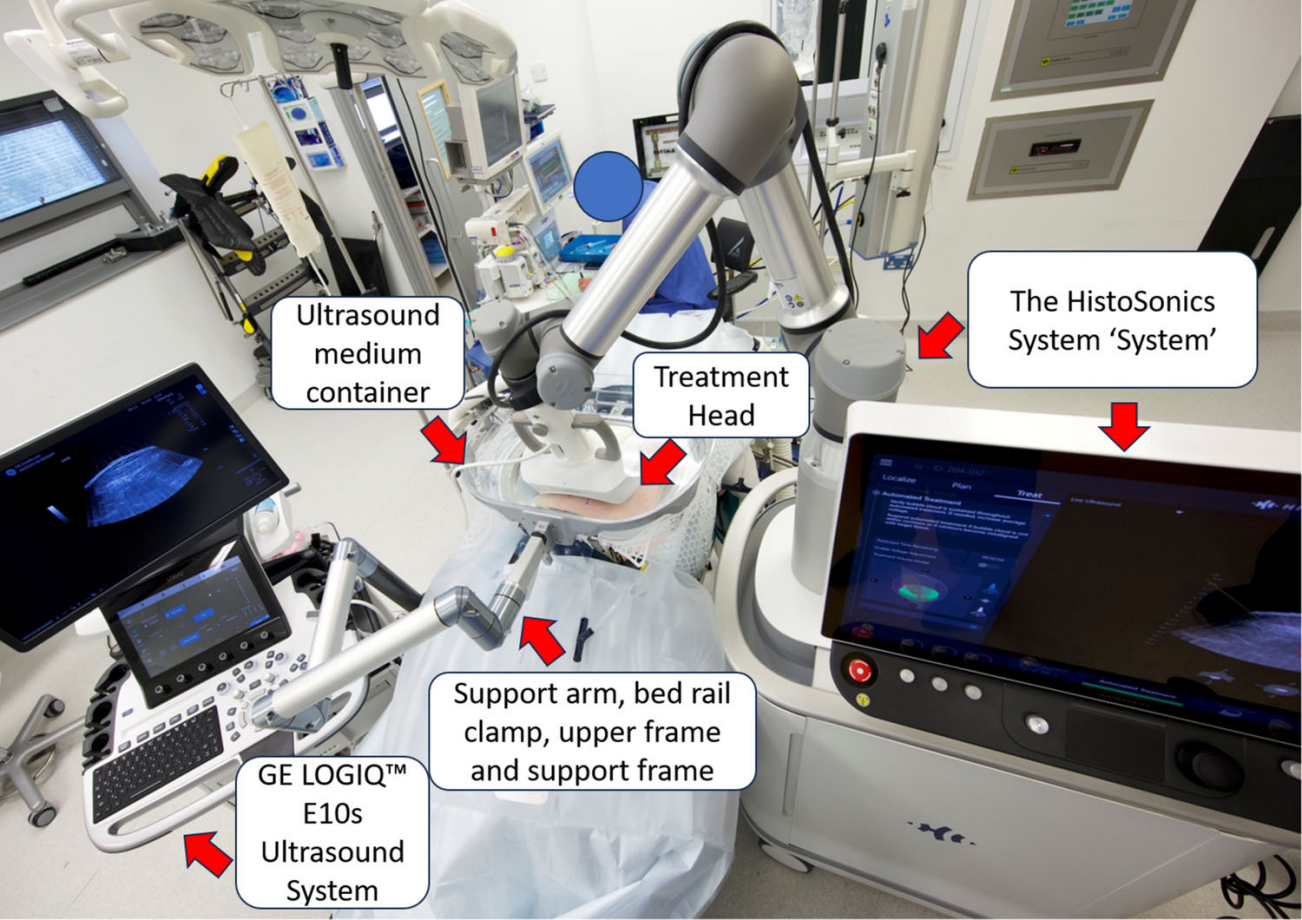
Helicopter view of the histosonics system components during procedure. The GE LOGIQ™ E10s Ultrasound System and probe are integrated into the HistoSonics System for tumour targeting, planning and monitoring treatment. The treatment head houses the histotripsy therapy transducer and an integrated ultrasound imaging probe. There are two treatment heads available for use depending on treatment depth (2–12 cm or 8–14 cm). The multi-joint articulating support arm secures on the bed rail and holds the reservoir containing ultrasound medium in the desired position above the patient. The patient membrane (single-use accessory) inserts into the frame to create the reservoir; it holds the ultrasound medium and conforms to the patient, acoustically coupling the treatment head to the patient

The HistoSonics System components shown in Fig. 1 are all non-sterile and reusable. A previous iteration of the device with the same mechanism of action was used at the beginning of the trial.

### Inclusion Criteria [13]

#### General Inclusion

1. Patient is ≥ 18 years of age.
2. Patient has signed the Ethics Committee approved trial Informed Consent Form prior to any trial related tests/procedures and is willing to comply with trial procedures and required follow-up assessments.
3. Patient is diagnosed with a non-metastatic solid renal mass ≤ 3 cm confirmed via CT or MRI ≤ 30 days prior to the index procedure date.
4. Patient can tolerate general anaesthesia.
5. Patient has an Eastern Cooperative Oncology Group Performance Status (ECOG PS) grade 0–2 at baseline screening.
6. Patient meets all the following functional criteria at ≤ 14 days prior to the planned index procedure date:
  White Blood Cell (WBC) ≥ 3,000/mm.^3^
  Absolute Neutrophil Count (ANC) ≥ 1,200/mm.^3^
  Haemoglobin (Hgb) ≥ 9 g/dL
  Platelet count ≥ 100,000/mm.^3^
  White Blood Cell (WBC) ≤ 40 cells/µL via urinalysis
  Albumin ≤ 300,000 mg/L via urinalysis
7. Patient has an eGFR ≥ 45 mL/min, ≤ 14 days prior to the planned index procedure date.
8. International Normalized Ratio (INR) score of < 1.5:
  If on anticoagulants, other than aspirin or non-steroidal anti-inflammatory drugs, assessment must be performed on the day of the procedure; OR
  If only on aspirin or non-steroidal anti-inflammatory drugs, assessment must be performed ≤ 14 days prior to the planned index procedure date; OR
  If not on anticoagulants, assessment must be performed ≤ 14 days prior to the planned index procedure date
9. Biopsy is required to determine the type of tumour and must be performed ≥ 14 days prior to the planned index procedure date.

#### Index-Procedure Ultrasound Imaging Inclusion (assessed day of procedure)

10. The tumour selected for histotripsy treatment must be ≤ 3 cm in longest diameter.
11. Patient has an adequate acoustic window to visualize targeted tumour using the HistoSonics System.
12. Patient will undergo histotripsy treatment of only one (1) tumour during the index procedure, regardless of how many tumours the patient has.

### Exclusion Criteria [13]

#### General Exclusion

1. Patient is pregnant or planning to become pregnant or nursing (lactating) during the trial period.
2. Patient is enrolled and being actively treated in another investigational pharmaceutical or device trial ≤ 30 days prior to planned index procedure date.
3. Patient is undergoing active chemotherapy for any cancer ≤ 14 days prior to planned index procedure date.
4. Patient is undergoing active immunotherapy ≤ 40 days prior to planned index procedure date.
5. In the Investigator's opinion, the patient has co-morbid disease(s) or condition(s) that would cause undue risk and preclude safe use of the HistoSonics System.
6. Patient is on dialysis or being considered for dialysis.
7. Patient has not recovered to Common Terminology Criteria for Adverse Events (CTCAE) grade 2 or better from any adverse effects (except alopecia and neuropathy) related to previous anti-cancer therapy.
8. Patient has an uncorrectable coagulopathy other than that induced by aspirin or non-steroidal anti-inflammatory drugs.
9. Patient has a planned cancer treatment (e.g., nephrectomy, chemotherapy, immunotherapy etc.) prior to completion of the 30-day follow-up visit.
10. Patient has had previous treatments with chemotherapy, radiotherapy, or both that have not been discontinued ≥ 14 days prior to the planned index procedure date and have not recovered (CTCAE grade 2 or better) from related toxicity (exclusive of alopecia and neuropathy).
11. Patient has previous treatment with immunotherapies that has not been discontinued ≥ 40-days prior to the planned index procedure date and has not recovered from related toxicity (CTCAE grade 2 or better).
12. Patient has a life expectancy less than one (< 1) year.
13. In the investigator's opinion, histotripsy is not a treatment option for the patient.
14. Patient has a concurrent condition that could jeopardize the safety of the patient or compliance with the protocol.
15. Patients' targeted tumour has had prior locoregional therapy (e.g., ablation, embolization, radiation).
16. Patients' tumour is not treatable by the System's working ranges (refer to User Guide).
17. In the physician's opinion, the anticipated risk of intervention outweighs the potential benefits of the intervention.
18. Patient has acute renal failure.
19. Patient has a genetic predisposition to kidney cancer such as:
  Von Hippel Lindau (VHL)
  Hereditary Papillary Renal Carcinoma (HPRC)
  Hereditary Papillary Renal Carcinoma (HPRC)
  Birt-Hogg-Dubé Syndrome (BHD)
  Tuberous Sclerosis Complex (TSC)
  Hereditary Leiomyomata's Renal Cell Carcinoma (HLRCC)
  Reed's Syndrome
  Succinate Dehydrogenase B Deficiency (SDHB)
  BRCA 1 associated protein -1 (BAP1) Renal Cell Carcinoma
  MITF predisposed Renal Cell Carcinoma
20. Tumour is an angiomyolipoma.
21. Patient has a known sensitivity to contrast media and cannot be adequately pre-medicated.

#### Index-Procedure Ultrasound Imaging Exclusion (assessed day of procedure)

22. The targeted tumour is not clearly visible with diagnostic ultrasound and either magnetic resonance imaging (MRI) or computerized tomography (CT).
23. Targeted tumour with adequate margin overlaps the renal pelvis, main renal vessel, ureter, or other vital structure.
24. Targeted tumour with adequate margin overlaps a non-targeted tumour visible via imaging.
25. The treatment of the tumour will not allow for an adequate margin as determined by the investigator.

### Statistics

An enrolment of up to 20 treated patients was selected to provide sufficient details on the efficacy and safety profile of the device. As a pilot trial, no formal hypotheses were defined; the endpoints are designed to provide additional information for the development of a pivotal trial. All endpoints will be summarized descriptively with 95% (Wilson) confidence intervals.

### Study Cohort and Follow-Up

Written site activation from the trial sponsor, including Ethics Committee (EC) approval of the CAIN protocol and Informed Consent Form (ICF), must be obtained prior to enrolling patients in the trial. The trial enrolment process is illustrated in. Patients who sign an ICF will be considered enrolled in the trial. A patient who does not meet inclusion/exclusion criteria prior to treatment will be exited. Patients will be considered treated once the investigator has delivered histotripsy (energy) to the patient.

Treated patients will have a follow-up visit performed at 14 days, 30 days, 90 days, and 180 days post-procedure.

### Histotripsy Treatment

**Fig. 2:**
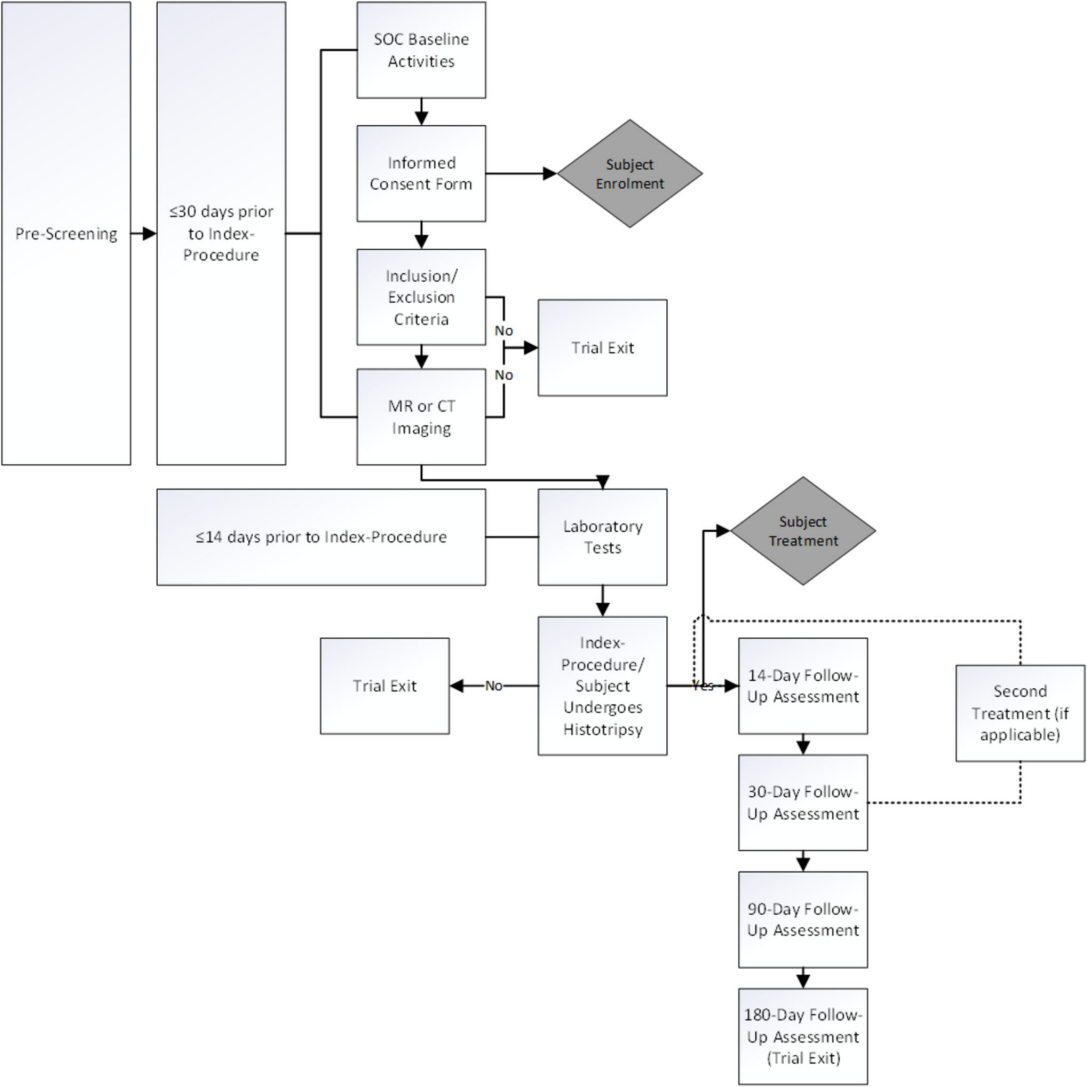
Trial enrolment

The trial investigator will be trained on the HistoSonics System and may be supported during the procedure by qualified and trained Sponsor personnel and/or designees. Patients will be placed under general anaesthesia to reduce patient discomfort and provide for investigator complete control, including patient breathing and motion during the procedure (Fig. 2).

**Fig. 3:**
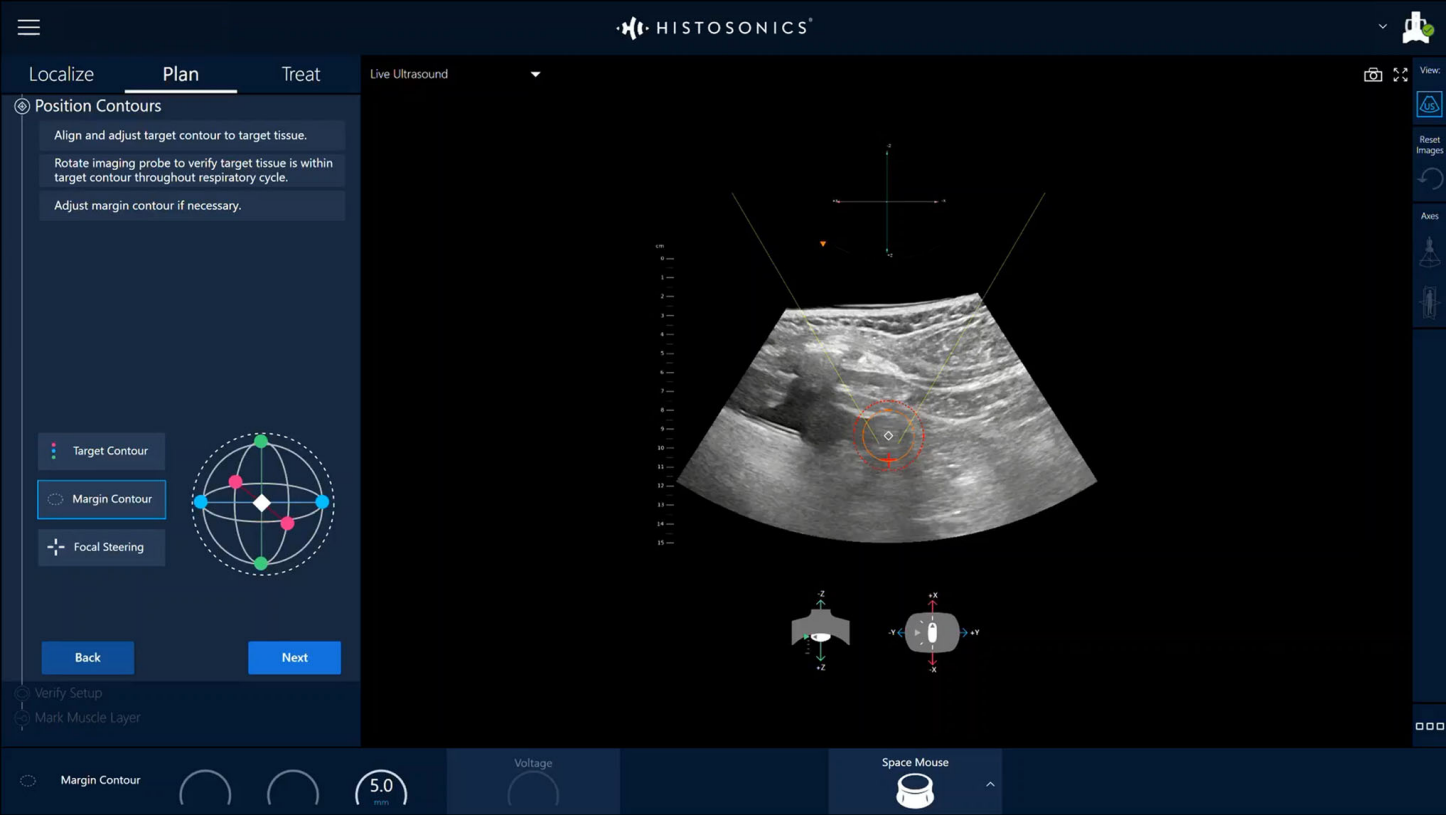
HistoSonics System User Interface. The HistoSonics System user interface provides the physician access to reference images and enables targeting, planning and treatment in real time. The workflow panel (displayed on the left) guides the physician during the localize, plan, and treat phases. The live ultrasound is displayed in the middle; using the workflow, the physician defines the PTV (target tumour [orange ellipse] and margin [red ellipse])

Once the patient is under general anaesthesia, the treatment head will be navigated via the robotic treatment arm of the HistoSonics System and the kidney tumour will be visualised using ultrasound (Fig. 3). Once the tumour is localized and the target tumour and desired margin are contoured, the investigator calibrates and establishes energy settings. The robotic arm navigates through the planned treatment volume (PTV) along the operational axes (x, y, z, pitch, roll, and yaw) and establishes the voltage settings at seven plan points required to achieve histotripsy cavitation throughout the entire PTV. Once treatment planning is completed, the treatment is executed via automatic treatment by the system with the pre-defined treatment time as stated on the system.

### Baseline, Pre- and Post-Procedural Assessments

Table 1 outlines the required tests and procedures.

**Table 1:** Summary of required tests and procedures

Procedure/Test/Data Collection	Pre-screen	Baseline	Pre- Index Procedure	Index Procedure/Treatment Procedure	Post-Index Procedure/ Discharge	14-Day Follow up Phone Assessment	30-Day Follow-up Visit	90-Day Follow-up Visit	180-Day Follow-up Visit
Visit Window		≤ 30 Days prior to Index Procedure	≤ 14 or ≥ 14 days	Data/ Values collected in procedure room	≤ 36 h	± 3 Days	± 3 Days	± 15 Days	± 30 Days
Informed Consent		×							
General Inclusion/Exclusion Criteria		×							
Index Procedure Imaging Inclusion/Exclusion Criteria	×			×					
Medical History with Demographics		×							
Physical Exam (Height/Weight)		×							
Biopsy			≥ 14 days						
R.E.N.A.L Nephrometry Score		×							
INR Assessment			≤ 14 days	×					
ECOG PS Grade		×					×	×	×
Imaging (CT/MRI)		×			×		×	×	×
Trial Enrolment				×					
Index Procedure Details				×					
Brief Pain Inventory (BPI)				×		×			
End of Trial									×
*Clinical Laboratory Tests*									
Blood Tests (CBC, INR value, basic metabolic panel, liver function test (albumin), serum creatinine)			≤ 14 days		×		×	×	×
Pregnancy Test (Urine or blood)			≤ 14 days						
Urinalysis			≤ 14 days		×		×	×	×
**Other**									
Adverse Event Assessment				×	×	×	×	×	×
Complaints/Device Deficiencies				×					
Protocol Deviation Assessment		×	×	×	×	×	×	×	×

### Outcome Measures [13]

The primary effectiveness endpoint is technical success, defined as complete coverage of the tumour by the histotripsy treatment zone as determined ≤ 36 h post-index procedure by contrast-enhanced CT or MR imaging, and will be assessed by the imaging core lab. The primary safety endpoint is freedom from index procedure related major complications, defined by Clavien-Dindo Classification [14] Grade 3 or higher up to 30 days after the last histotripsy procedure. Retreatment with histotripsy is not considered a major complication (retreatment can be completed ≤ 30 days of index procedure).

The secondary endpoints are technique efficacy (primary), defined as the percentage of targeted tumours successfully eradicated post-index procedure assessed via MRI or CT at 90-days post-index procedure without repeat histotripsy, and technique efficacy (secondary), defined as the percentage of targeted tumours successfully eradicated post-index procedure assessed via MRI or CT at 90-days post-index procedure after repeat histotripsy. Both secondary endpoints will be assessed by the imaging core lab.

### Trial Status

Twenty patients were enrolled in the CAIN trial between March 2023 and November 2024 at Leeds Teaching Hospitals NHS Trust, Leeds, UK. Primary endpoint results are expected to be presented in 2025, and 6-month results are expected to be presented in 2026.

## Discussion

As with any new therapy, there is a learning curve when translating and adopting new innovative technology. Histotripsy is a non-invasive and non-thermal technology and uses externally delivered energy to treat tumours without breaching the skin surface. As such, histotripsy differs from conventional Interventional Oncology (IO) procedures for renal tumour treatment, which typically involve percutaneous insertion of electrodes/antennas (heat-based energy) or cryoprobes (ice-based energy) under imaging guidance.

To ensure successful recruitment and trial delivery, the clinical trial delivery team will need to ensure meticulous patient selection and robust patient assessment at the outpatient consultant clinic with ultrasound scanning capabilities to ensure the renal tumour intended for treatment is well visualized under ultrasound with the patient in the planned position for treatment and throughout the respiratory cycle. Following consent, pre-procedural planning is facilitated by a software tool developed by HistoSonics that allows the operator to simulate treatment plans. In addition, pre-procedural discussion with technical support and a peer group support team ahead of the histotripsy treatment is vital in determining optimal positioning and therapy system approach based on the baseline cross-sectional imaging acquired.

On the day of the procedure, it is vital to brief the team in the theatre suite regarding the intra-procedural patient positioning as well as effective breathing and motion management to ensure optimal delivery of the histotripsy treatment. Therefore, it is critical to work with anaesthesia colleagues to minimize motion (e.g., by reducing tidal volumes) to prevent the tumour from migrating out of the planned treatment volume.

From the operator perspective, one of the crucial aspects for successful delivery of the treatment is to ensure the patient is positioned with the shortest route from treatment head to the target tumour with minimal angulation of the treatment head. The availability of the surgical theatre bed with the option of ‘breaking’ the bed to open up the flank can be advantageous to ensure best visualization and direct targeting of the tumour. Vital components of this novel treatment, including patient positioning, visualization under ultrasound, and motion management directly contribute to the success of treating kidney tumours with histotripsy.

Currently there are multiple local therapies for small renal tumours. The successful implementation of a non-invasive treatment that avoids thermal-related collateral damage, particularly to central renal structures, would be a significant clinical advancement. The CAIN feasibility trial results will be published and disseminated in a peer reviewed scientific journal and will provide further understanding into technology advancement in renal tumour treatment and facilitate future clinical trial designs.
